# Association of *IFITM3* rs12252 polymorphisms, BMI, diabetes, and hypercholesterolemia with mild flu in an Iranian population

**DOI:** 10.1186/s12985-017-0884-4

**Published:** 2017-11-09

**Authors:** Parvaneh Mehrbod, Sana Eybpoosh, Fatemeh Fotouhi, Hadiseh Shokouhi Targhi, Vahideh Mazaheri, Behrokh Farahmand

**Affiliations:** 1 Influenza and Other Respiratory Viruses Department, Pasteur Institute of Iran, Tehran, Iran; 2 Department of Epidemiology and Biostatistics, Research Centre for Emerging and Reemerging Infectious Diseases, Pasteur Institute of Iran, Tehran, Iran

**Keywords:** Human influenza, IFITM3 protein, Body mass index, Hypercholesterolemia, Diabetes, Polymorphism, Iran

## Abstract

**Background:**

*IFITM3* has been suggested to be associated with infection in some ethnic groups. Diabetes and hypercholesterolemia are also important clinical conditions that can predispose individuals to infection. The aim of this study was to investigate the association of rs12252 C polymorphism, BMI, diabetes, and hypercholesterolemia with mild flu in an Iranian population.

**Methods:**

We conducted a case-control study, including 79 mild flu and 125 flu-negative individuals attending primary care centers of three provinces of Iran (*i.e,* Markazi, Semnan, and Zanjan). Pharyngeal swab specimens were collected from all participants, and were subjected to RNA and DNA extractions for Real-time PCR and PCR tests. All PCR products were then sequenced to find T/C polymorphisms in the rs12252 region. Data on demographic, anthropometric, and clinical variables were collected from participants’ medical records available in the primary care centers. The data was analyzed using DNASIS (v. 2.5) and Stata (v.11) software.

**Results:**

All participants were of Fars ethnic background. The allele frequency for rs12252-C was found to be 9.49% among cases and 2.40% among controls. Carriers of the rs12252 C allele (CT + CC genotypes) showed 5.92 folds increase in the risk of mild flu comparing to the T allele homozygotes (*P* value: 0.007). We also found a significant positive association between rs12252 C allele heterozygote and mild flu (OR: 7.62, *P* value: 0.008), but not in C allele homozygote group (OR: 2.71, *P* value: 0.406). Similarly, we did not find a significant association between mild flu and BMI (OR: 1.06, *P* value: 0.087), diabetes (OR: 0.61, *P* value: 0.392), and hypercholesterolemia (OR: 0.50, *P* value: 0.393) in multivariable logistic regression.

**Conclusions:**

This is the first study evaluating the association between rs12252 polymorphisms, diabetes, hypercholesterolemia, and BMI and susceptibility to mild flu in an Iranian population. Our results suggest a significant positive association between mild flu and rs12252 C allele heterozygous and carriage. Future replication of the strong association observed here between rs12252 C allele carriage and mild flu might candidate this polymorphism as a genetic marker for early screening of susceptibility to mild flu. Lack of significant association between C allele homozygous and mild flu, observed in this study, might be the result of small sample size in this group.

**Trial registration:**

IR.PII.REC.1395.3.

**Electronic supplementary material:**

The online version of this article (10.1186/s12985-017-0884-4) contains supplementary material, which is available to authorized users.

## Background

Influenza is the result of both host and viral genetic components combination. The viral genetic determinants and host immunity have been widely considered, while the host genetic determinants remain elusive [13]. The study of the host genetic factors involved in susceptibility to influenza is a promising strategy that may identify potential therapeutic targets [18]. As a result, systematic investigation of the association between host genetic factors and the occurrence of influenza is of pivotal importance. In 2009, the World Health Organization (WHO) identified as priority the studies which considered the role of host genetic factors on susceptibility to severe flu [17].

During viral infection, viral proteins and viral nucleic acids are detected by pathogen recognition receptors (PRRs). These recognition patterns activate signaling transduction pathways that trigger production of type I interferon and other related cytokines [3, 25]. Type I interferon then elevates the expression of hundreds of genes, named interferon stimulated genes (ISGs), that weaken virus replication by a variety of mechanisms [3, 25].

It has been suggested that the expression of interferon-inducible transmembrane (*IFITM*) genes restrict the replication of several highly pathogenic human viruses, especially those that enter the cell via acidic endosome [3, 10, 26]. These viruses include severe acute respiratory syndrome (SARS), coronavirus, Marburg and Ebola viruses, influenza A viruses (IAVs), dengue virus, and HIV-1 [2, 3, 5, 14, 20].

In humans, it has been predicted that rare SNP rs12252-C allele of *IFITM3* produces an alternatively spliced transcript that encodes a truncated protein (Δ21 IFITM3) leading to reduced control of virus replication in vitro. However, expression at mRNA or protein level of the Δ21 IFITM3 variant has not been detected to date [7, 9].

The value of *IFITM3* in influenza virus infection was determined by Everitt et al. who showed that severity of influenza virus infection was greatly increased in *IFITM3* knockout mice compared to the wild-type animals [9]. Everitt et al. and Zhang et al. demonstrated increased morbidity and mortality in rs12252-C homozygotes during the H1N1 influenza pandemic. Due to the high frequency of the rs12252-C allele in Asians, the effect of the homozygosity for this allele was proposed to translate to a population-attributable risk of 54.3% for severe flu in the Chinese population compared with 5.4% in Northern Europeans. Both studies were performed with small sample sizes of H1N1-infected patients, calling for further studies on large sample sizes. Two studies reported a strong association between *IFITM3* SNP rs12252-C and worse clinical outcomes in patients infected with 2009 IAV pandemic. The studies, however, doubt the presence of such association, at least among Europeans, as several rs-12,252-C homozygous patients only developed a mild flu [9, 31].

It appears that IFITM3 inhibits an early event after endocytosis of virion particles, by blocking virus release into the cytosol [1, 10, 28]. IFITM3 protein is considered to be the most active against IAV and resides in the late endosomes and lysosomes using several orthologous genetic approaches [3, 14]. Wang and colleagues showed that inflammatory immune responses linked to the C/C *IFITM3* genotype during H7N9 infection play an important role in the pathogenesis of influenza. They found that patients with rs12252-C/C IFN-*IFITM3* genotype had more rapid disease progression, and were less likely to survive [27].

Although previous studies concluded that the rs12252-C variant leads to reduced control of virus replication in vitro, posterior studies did not find such effects. For example, Mills et al. found an association between rs12252 rare allele C/C homozygotes and susceptibility to mild flu but they could not confirm previous reports for the association between this polymorphism and susceptibility to severe H1N1 infection [22]. The data obtained by López-Rodríguez et al. did not either suggest any role of rs12252-C in the development of severe IAV in their studied population. They suggested these groups might be at risk of severe influenza, hence individualized measures in the case of IAV is required [19].

Giao et al. also showed that except H1N1 influenza (pdm09), the risk of hospitalization due to other respiratory infections was higher among rs12252 C allele carriers comparing to patients with TT genotype [11].

It has been reported that local accumulation of cholesterol to the plasma membrane is important for clustering of viral structural proteins in lipid rafts to help assemble the virus particles [1]. Thus, the imbalance in cholesterol level may play a role in the susceptibility to influenza. The *IFITM3* gene mutation may lead to an increase in impaired cholesterol levels [1]. As an instance, mutation rate of *IFITM3* gene is shown to be high among Han Chinese population. Coronary artery disease and ischemic stroke are highly associated with BMI and serum cholesterol levels, and are the leading causes of morbidity and mortality in Han Chinese population [8, 12, 23]. Therefore, we performed this study to identify the association between mild flu and *IFITM3* rs12252-C polymorphism, BMI, diabetes and hypercholesterolemia.

## Methods

### Ethics statement

Written informed consent was obtained from all participants. All procedures performed in this study involving human participants were according to the principles Helsinki declaration. The research protocol was approved by the Ethics Committee of Pasteur Institute of Iran (Ethics code: IR.PII.REC.1395.3).

### Participants

We performed a case–control study on the samples collected from March 2015 to December 2015 in three provinces of northern-central Iran, including Markazi, Semnan, and Zanjan. Cases would be included in this study if they had apparent symptoms of respiratory tract infection, and were confirmed as mild flu with PCR test. Controls were flu-negative individuals based on the PCR results, and would be included in the study if they had no history of influenza. Both cases and controls should be based and currently residing in Markazi, Semnan, or Zanjan provinces. Having respiratory comorbidities, consumption of immune suppressive drugs, and previous vaccination against influenza virus were our exclusion criteria.

### Sample collection

Demographic and anthropometric characteristics measured for each participant included participants’ age (in years), province (Markazi, Semnan, and Zanjan provinces), Body Mass Index (BMI, kg/m^2^), diabetes (Yes/No), hypercholesterolemia (Yes/No), and genotype in the *IFITM3* rs12252 locus. The BMI of each participant was calculated using the formula: BMI = [weight (kg)] / [height (m)]^2^. The diabetes and hypercholesterolemia positive and negative cases were confirmed based on the patients’ history reports. To test for influenza, pharyngeal specimen from each individual was obtained with a Dacron swab. Specimens were collected on dry ice in transport media containing Pen/Strep and Amphotericin B, and then were transferred to Pasteur Institute of Iran for determination of IAV. All samples were stored at 4 °C for 24 h. Following vortex, swabs were discarded and the supernatants were aliquoted in sterile labeled micro-tubes and stored at −80 °C for further testing.

### Detecting IAV in swab samples

#### Viral RNA extraction

For each sample, viral RNA was extracted from 200 μl fluid specimen using High Pure Viral RNA Kit, according to the manufacturer’s instructions (Roche, Switzerland). The extracted RNA was isolated and re-suspended in 50 μl Elution Buffer and stored at −80 °C for Real-time PCR assay.

#### Primers and probes design

In quantitative real-time PCR (qRT-PCR), primers and probes were designed and synthesized by SinaClon Co. (Iran) based on the latest WHO guideline. Table 1 describes the primers used for amplification of viral genes and RNaseP as housekeeping control.

**Table 1:** Real-time PCR Primers and probes specifications

Primers/Probes	Sequence (5′ > 3′)	Working Concentration
InfA Forward	GAC CRA TCC TGT CAC CTC TGA C	40 μM
InfA Reverse	GCA TTY TGG ACA AAK CGT CTA	40 μM
InfA Probe^a^	TGC AGT CCT CGC TCA CTG GGC ACG	10 μM
SW InfA Forward	GCA CGG TCA GCA CTT ATY CTR AG	40 μM
SW InfA Reverse	GTG RGC TGG GTT TTC ATT TGG TC	40 μM
SW InfA Probe^b^	CYA CTG CAA GCC CA“T” ACA CAC AAG CAG GCA	10 μM
Flu B Primer^a^ M	GAGACACAATTGCCTACCTGCTT	10 pm
Flu B Primer^b^ M	TTCTTTCCCACCGAACCAAC	10 pm
B Probe M	AGAAGATGGAGAAGGCAAAGCAGAACTAGC	5 pm
Rnase P Forward	AGATTTGGACCTGCGAGCG	40 μM
Rnase P Reverse	GAGCGGCTGTCTCCACAAGT	40 μM
Rnase P Probe^a^	TTCTGACCTGAAGGCTCTGCGCG	10 μM

^a^TaqMan probes are labeled at 5′- end with the reporter molecule 6- carboxyfluorescein (FAM) and with the quencher Blackhole Quencher 1 (BHQ1) at the 3′- end

^b^TaqMan probes are labeled at 5′- end with the reporter molecule 6- carboxyfluorescein (FAM) and quenched internally at a modified “T” residue with BHQ1 with a modified 3′- end to prevent probe extension by Taq polymerase

#### One-step quantitative real-time PCR

Real-time PCR reactions were performed in total volume of 20 μl, using Rotor-Gene Q (QIAGEN). The reaction mixture consisted of 2X Master Mix, SuperScript^III^ RT/Platinum Taq Mix, specific primers and fluorescent probes. The thermal cycling program was followed based on WHO guideline. Briefly, reverse transcription was carried out at 50 °C for 30 min, followed by 95° for 2 min Taq inhibitor activation, and amplification at 95 °C for 15 s followed by 55 °C for 30 s.

### Genotyping of human *IFITM3* rs12252

#### Human DNA extraction

For each sample, genomic DNA was extracted from 100 μl fluid specimen using DNP™ High yield DNA Purification Kit, according to the manufacturer’s instructions (Cinnagen, Iran). Briefly, 400 μl of Lysis Solution was added to each sample. Following vortex and addition of precipitation solution, a second round vortex was performed. The samples were then centrifuged. Wash buffer was used for two times to wash the precipitation. Finally, solvent buffer was added and the tubes were warmed at 65 °C for 5 min. The supernatant, which was the purified DNA, was stored at −20 °C for PCR assay.

#### Primers design

Primers for PCR were designed and synthesized by SinaClon Co. (Iran) based on previous published articles [31]. The primers used for the amplification of human *IFITM3* rs12252 were as follow: *IFITM3*-F 5′-GGAAACTGTTGAGAAACCGAA-3′ and *IFITM3*-R 5′-CATACGCACCTTCACGGAGT-3′ to produce 351 bp PCR product. The18 S rRNA was amplified as internal housekeeping using theses primers sequences: 18 S-F 5′-GGCCCTGTAATTGGAATGAGTC-3′ and 18 S-R5′- CCAAGATCCAACTACGAGCTT-3′ to produce 145 bp PCR product.

#### Conventional PCR

PCR reaction was performed in total volume of 25 μl, using Thermocycler (Germany). The reaction mixture consisted of Taq DNA Polymerase 2× Master Mix RED with 1.5 mM MgCl_2_ (Amplicon, Denmark), specific primers and DNase-free water. Amplification was carried out at 95 °C for 30 s in order to pre-denature the template DNA, followed by 40 cycles of denaturation at 95 °C for 30 s, annealing at 60 °C for 1 min and extension at 72 °C for 1 min. The final extension was completed at 72 °C for 5 min. The PCR products were purified using PCR purification kit (Millipore), and subjected to sequencing (First Base Co, Malaysia) using the BigDye® Terminator v3.1 cycle sequencing kit chemistry. Then they were loaded into DNA Analyzer (GATC Biotech). Both sense and antisense strands of PCR products were directly sequenced using the same primers as the PCR amplification. SNPs were detected by direct sequence analysis using DNAsis MAX.3 software. The reference sequence for the *IFITM3* gene was based on the sequence of human chromosome 11, clone GRCh38.p2.

### Genetic and statistical analysis

Hardy-Weinberg equilibrium (HWE) was tested in cases and controls using the web tool developed by Rodriguez, and colleagues [24]. Using this tool, allele frequency and genotype distribution were calculated for cases and controls, as well. Statistical analyses were performed using Stata software (version 11). Continuous and categorical data were described in the usual fashion; means ± SDs were used to describe continuous variables, and numbers (percentages) were used to describe categorical variables. Association between categorical variables, including study group (mild flu vs. flu-negatives), rs12252 genotype (CC, CT, TT, and C carriers), rs12252 allele (C and T), province (Markazi, Semnan, and Zanjan), diabetes (positive vs. negative), and hypercholesterolemia (positive vs. negative), was assessed using Pearson’s chi-squared test. For each cell with zero counts in contingency tables, Yates’s correction was used. Also, where more than 20% of cells had counts less than 5, the alternative Fisher’s exact test was used instead of Pearson’s chi-squared test. Univariable logistic regression was used to test the association of each independent variable with mild flu. Variables with a *P* value smaller than 0.2 in the univariable model were included into the multivariable logistic regression. Also, a backward method using likelihood ratio tests (LRT) was used to make the model as simple as possible. As we sampled cases and controls from three distinct provinces of Iran, we retained the variable “province” in the final model. This was to ensure that residual population admixture, due to differences in participants’ area of residence, does not confound the association between rs12252 genotypes and mild flu. All statistical tests were two-tailed with a type I error of 0.05. Observed powers of the estimated crude and adjusted odds ratios (OR) were also calculated to assess the robustness of our results (Additional file 1).

## Results

### Participants

A total of 79 mild flu cases and 125 controls were included in this study. Participants’ mean age in the case and control groups was 46.46 (SD: 22.90) and 50.62 (SD: 25.30) years, respectively (*P* value _t-test_: 0.275). All participants were of Fars ethnicity, which is the dominant ethnicity in Iran. The distribution pattern of flu-positive and flu-negative individuals was similar across three provinces, with Semnan having the highest number of both cases (45.57%) and controls (72.80%), and Zanjan having the lowest number of cases (12.66%) and controls (7.20%). The difference in the number of cases and controls was significant between Semnan and Zanjan (OR_Seman vs. Zanjan_: 0.36, *P* value: 0.039), but not between Semanan and Markazi or between Zanjan and Markazi provinces. Details of participants’ characteristics are shown in Table 2.

**Table 2:** Participants’ characteristics and univariable logistic regression of the association between mild flu and rs12252 polymorphisms, BMI, diabetes, and hypercholesterolemia in an Iranian sample with Fars ethnic background

Variables	Case	Control	OR	95% CI	*P* value
	*n* = 79	*n* = 125			
Age _(mean ± SD)_	46.46 ± 22.90	50.62 ± 25.30	0.99	0.98, 1.00	0.275
BMI _(mean ± SD)_	26.51 ± 8.67	23.76 ± 4.67	1.06	1.01, 1.13	0.034
Province _n (%)_					
Semnan	36 (45.57)	91 (72.80)	0.36	0.13, 0.95	0.039
Markazi	33 (41.77)	25 (20.00)	1.19	0.42, 3.36	0.745
Zanjan	10 (12.66)	9 (7.20)	1	–	–
Hypercholesterolemia _n (%)_					
Positive	3 (3.80)	16 (12.80)	0.27	0.08, 0.96	0.042
Negative	76 (96.20)	109 (87.20)	1	–	–
Diabetes _n (%)_					
Positive	12 (15.19)	42 (33.60)	0.35	0.17, 0.73	0.005
Negative	67 (84.81)	83 (66.40)	1	–	–
Genotype _n (%)_					
CC	3 (3.80)	1 (0.80)	5.37	0.55, 52.68	0.149
TC	9 (11.39)	4 (3.20)	4.03	1.20, 13.58	0.025
CC + CT	12 (15.19)	5 (4.00)	4.29	1.45, 12.72	0.008
TT	67 (84.81)	120 (96.00)	1	–	–
Allele _n (%)_					
C allele	15 (9.49)	6 (2.40)	4.26	1.51, 13.67	0.0016
T allele	143 (90.51)	244 (97.60)	1	–	–

### Anthropometric and clinical characteristics of study participants

The mean BMI value was slightly higher among mild flu patients rather than in controls (*P* value: 0.034). Prevalence of hypercholesterolemia and diabetes was also significantly higher in controls rather than in mild flu patients (*P* values: 0.042 and 0.005, respectively; Table 2).

### Genotyping and genetic analysis

The TT genotype was the most prevalent genotype both among cases (84.81%) and controls (96.00%). Similarly, the CC genotype was the least prevalent genotype in both groups (cases: 3.80%, controls: 0.80%, Table 2). In both groups, the alleles were in HWE (*P* value > 0.05, Table 3). The distribution of TT, TC, CC genotypes as well as C carriers was not significantly different across three provinces, either in total, or separately among cases and controls (Table 3).

**Table 3:** *IFITM3* rs12252 genotype frequencies among mild flu cases and flu-negative controls, based on participants’ province of residence

	Genotype					HWE	
	CC	TC	TT	*P* value^a^*	*P* value^b^*	X^2^	*P* value
Total				0.120	0.926	–	–
Province							
Zanjan	0	1	18				
Semnan	1	11	115				
Markazi	3	1	54				
Cases						0.63	> 0.05
Province				0.073	0.099	–	–
Zanjan	0	0	10				
Semnan	1	8	27				
Markazi	2	1	30				
Controls						0.03	> 0.05
Province				0.185	0.361	–	–
Zanjan	0	1	8				
Semnan	0	3	88				
Markazi	1	0	24				

*HWE* Hardy Weinberg Equilibrium

*Fisher’s exact test

^a^Association analyses are performed based on codominant genetic model (CC, TC, and TT)

^b^Association analyses are performed based on dominant genetic model (CT + CC vs. TT)

### Association of rs12252 polymorphisms, BMI, diabetes, and hypercholesterolemia with mild flu

Results of the univariable logistic regression showed that having TC genotype rather than TT, significantly increased the risk of mild flu (OR: 4.03, *P* value: 0.025). The same effect, but with greater magnitude, was also observed for the CC genotype rather than the TT genotype, however, this effect was not statistically significant (OR: 5.37, *P* value: 0.149; Table 2). In the final model, the effect of both TC and CC genotypes on the risk of mild flu showed an increase, but again this effect was only significant in the TC group. Furthermore, carriers of the rs12252 C allele (CT + CC genotypes) showed 5.92 folds increase in the risk of mild flu comparing to the T allele homozygotes (*P* value: 0.007). In the final model, the effect of BMI, diabetes, and hypercholesterolemia were no longer statistically significant (Table 4). Details of multivariable regression process are presented in Additional file 2.

**Table 4:** Multivariable logistic regression of the association between *IFITM3* rs12252 polymorphisms and mild flu in an Iranian sample with Fars ethnic background

Genotype	OR^a^	95% CI	*P* value
CC	2.71	0.26, 28.57	0.406
CT	7.62	1.69, 34.39	0.008
CC + CT	5.92	1.59, 22.09	0.007
TT	1	–	–

^a^ORs are adjusted for BMI and province of residence

## Discussion

Advances in the genomic area have enlightened the role of genetic factors in susceptibility to various infectious diseases. In this regard, IFITM3 protein encoded by *IFITM* gene, have shown to be associated with susceptibility to several viral infections, such as West Nile virus, dengue virus, rhinovirus, corona virus, HIV, respiratory syncytial virus, and influenza A virus [3, 15, 20, 28]. In case of influenza infection, however, it is not clear whether the reported association relates to the infection severity.

In this study, we investigated the association between mild flu and *IFITM3* rs12252 variant, BMI, diabetes, and hypercholesterolemia in an Iranian population. Our results suggest a significant positive association between mild flu and rs12252 C allele heterozygous (CT) and carriage (CT + CC), but not between mild flu and C allele homozygous (CC), BMI, diabetes, and hypercholesterolemia.

IFITM/ IFITM3 proteins are membrane-associated proteins, and directly interfere with the virus entry into the host’s cells [20]. Since virus entry often proceeds through endocytosis, it is possible that IFITM proteins are involved in endocytosis, by detecting and eliminating viral invaders before establishment of infection [3, 21]. Therefore, mutation in the gene coding for these proteins can lead to susceptibility to various infections. Yount, et al. (2010) introduced *IFITM3* in mouse dendritic cells. They mentioned S-palmitoylation of IFITM3 on proximal cysteines controls its clustering in membrane compartments and its antiviral activity against influenza virus. [30]. Bailey and colleagues also found that IAV lung titers were higher in *Ifitm3* knockout mice versus wild-type animals [2].

The association of *IFITM* gene polymorphisms with mild and sever influenza has been investigated in different ethnicities. For example, Zhang, et al. pointed to the significant role of *IFITM3* genetic variants on the epidemiology of influenza among Chinese individuals [31]. Yang, et al. found a significant association between *IFITM3*-SNP rs12252 and susceptibility to severe, but not mild flu, among Asian and Caucasian ethnicities [29]. Conversely, Mills, et al. found evidence of an association between rs12252 C allele homozygotes and susceptibility to mild flu in patients with Caucasian ancestry, attending primary care, but could not confirm the association between this SNP and susceptibility to severe flu [22]. Studies conducted by López-Rodríguez and Giao, et al. did not find evidence for the association between rs12252 C allele homozygotes and susceptibility to sever flu [11, 19, 22].

Based on the conclusion from the review paper of Ciancanelli et al. which mentioned IFITM3 SNP could not be a major causal factor, but perhaps a modifier, of the outcome of influenza infection [6], we also concluded there is only convincing experimental evidence for the role of wild-type IFITM3 but further work is required to define the role of IFITM3 SNPs as modifiers for severe influenza. The inconsistent results reported by studies investigated the association between *IFITM3* SNP rs12252 and influenza, made Yang, et al. to conduct a systematic review. Their meta-analysis included 445 patients with influenza and 4180 controls from four studies, including different ethnic and severity subgroups. Their results showed that patients with the rs12252-C/C genotype were more likely to increase the susceptibility to severe but not in mild flu, in both UK Caucasians and Han Chinese [29].

Our results also showed that rs12252-C carriers have an increased risk of mild flu rather than patients with TT genotype. This may be explained as due to the differences in participants’ ethnic background, since we studied Iranian ethnicities while Yang, et al. focused on UK Caucasians and Han Chinese ethnicities. Although, the rs12252-C allele in the Iranian population was rare, the overall effect of this allele on the risk of influenza was found to be considerably high. As our sample size was small, the power for some of our estimates was not desirable. In case such findings being replicated by future studies, the knowledge can be used in effective management of the epidemics. For example, understanding the biological pathways leading to mild and severe flu may lead to improvement of therapeutic options, and can alter existing vaccination practices. Findings of large-scale studies on genetic susceptibility to influenza would also guide population-based prevention efforts. For example, by considering the genetic susceptibility of different sub-populations to influenza virus in a country, human and medical resources would be allocated to high risk areas in case epidemics/pandemics occur.

The *IFITM3* allele frequency varies significantly among different ethnicities. For example, the frequency of rs12252 C allele homozygotes in Han Chinese, Japanese, Northern Europeans, and Englanders has been shown to vary greatly, from 0% among Japanese to 44% among Chinese populations (25, 44, 0 and 2%, respectively) [16]. Little is known about the frequency of this genotype in Iran. In our study, the C allele frequency observed among flu-negative individuals was 2.40%, resembling the allele frequency among Englanders. Studies with larger sample sizes would provide a better understanding about the frequency of susceptibility alleles in Iran.

In this study, we recruited our samples from three provinces of Northern-Central Iran, including Markazi, Semnan, and Zanjan provinces. Although all participants were of Iranian ethnicity, participants of each province might be different regarding their genetic and social history, leading to residual confounding due to population stratification. Population stratification is related to situations when cases and controls are of different ethnic backgrounds with different genetic compositions, and has been noted as the most important type of the bias in genetic association studies. To control for the effect of residual population admixture on our results, we adjusted genetic association between *IFITM3*-SNP rs12252 and susceptibility to mild flu for participants’ residence area. Growing empirical and theoretical evidence suggests that statistical adjustment for confounding effect of ethnicity provides estimates of genetic associations which are robust to bias from population stratification [4].

In our study, we did not observe a significant association between rs12252 C allele homozygote, BMI, diabetes, and hypercholesterolemia and mild flu. This might be due to relatively small sample size in our study, which led to moderate power of some parameter estimates. To verify these findings, further studies on the Iranian ethnicity with larger sample sizes are recommended.

## Conclusions

To the best of our knowledge, this is the first study evaluating the association between IFITM3 rs12252 polymorphism, diabetes, hypercholesterolemia and BMI with susceptibility to mild flu in an Iranian sample. Our results suggest a significant positive association between mild flu and rs12252 C allele heterozygous and carriage. However, we did not observe an association between mild flu and rs12252 C allele homozygous, BMI, diabetes, and hypercholesterolemia. Further studies are recommended to verify these findings in a larger sample of Iranian individuals. Future replication of the strong association observed here between rs12252 C allele carriage and mild flu would be beneficial in candidating this polymorphism as a genetic marker for early screening of susceptibility to mild flu.

## Additional files

Additional file 1: Multivariable logistic regression of the association between *IFITM3* rs12252 polymorphisms and mild flu in an Iranian sample with Fars ethnic background: Intermediate results. (DOCX 16 kb)

Additional file 2: Univariable and multivariable logistic regression of the association between mild flu and *IFITM3* rs12252 polymorphisms, BMI, diabetes, and hypercholesterolemia in an Iranian sample with Fars ethnic background: observed powers. (DOCX 20 kb)

## Abbreviations

IAV: Influenza A virus
IFITM: Interferon-inducible transmembrane
ISGs: Interferon stimulated genes
PRRs: Pathogen recognition receptors
qRT-PCR: Quantitative real-time PCR
SARS: Severe acute respiratory syndrome
WHO: World Health Organization
