# Supplementary material for: Association of IFITM3 rs12252 polymorphisms, BMI, diabetes, and hypercholesterolemia with mild flu in an Iranian population
Source: Virol J. 2017 Nov 9;14:218. doi: 10.1186/s12985-017-0884-4 (PMC5680824; doi:10.1186/s12985-017-0884-4)
Supplement: Supplementary file 1 — Multivariable logistic regression of the association between IFITM3 rs12252 polymorphisms and mild flu in an Iranian sample with Fars ethnic background: Intermediate results. (DOCX 16 kb) [file 12985_2017_884_MOESM1_ESM.docx]

**Additional File 1.** Multivariable logistic regression of the association between *IFITM3* rs12252 polymorphisms and mild flu in an Iranian sample with Fars ethnic background: Intermediate results

| **Variable** | **Adjusted OR** | **95% CI** | ***P* value** | **Final model** | **95% CI** | ***P* value** |
| --- | --- | --- | --- | --- | --- | --- |
| **Age** | 0.96 | 0.80, 1.16 | 0. 653 | - | - | - |
| **Province** |  |  |  |  |  |  |
| Markazi | 0.83 | 0.24, 2.90 | 0.766 | 1.18 | 0.41, 3.39 | 0.756 |
| Semnan | 0.21 | 0.07, 0.65 | 0.007 | 0.31 | 0.12, 0.86 | 0.024 |
| Zanjan | 1 | - | - | 1 | - | - |
| **Genotype** |  |  |  |  |  |  |
| CC | 1.20 | 0.09, 16.11 | 0.885 | 2.71 | 0.26, 28.57 | 0.406 |
| CT | 7.95 | 1.71, 37.06 | 0.008 | 7.62 | 1.69, 34.39 | 0.008 |
| CC+CT | 5.41 | 1.37, 21.28 | 0.016 | 5.92 | 1.59, 22.09 | 0.007 |
| TT | 1 | - | - | 1 | - | - |
| **BMI** | 1.10 | 0.99, 1.19 | 0.050 | 1.06 | 0.99, 1.12 | 0.087 |
| **Hypercholesterolemia** | 0.50 | 0.20, 1.88 | 0.393 | - | - | - |
| **Diabetes** | 0.61 | 0.20, 1.88 | 0.392 | - | - | - |
