# Supplementary material for: Association of IFITM3 rs12252 polymorphisms, BMI, diabetes, and hypercholesterolemia with mild flu in an Iranian population
Source: Virol J. 2017 Nov 9;14:218. doi: 10.1186/s12985-017-0884-4 (PMC5680824; doi:10.1186/s12985-017-0884-4)
Supplement: Supplementary file 2 — Univariable and multivariable logistic regression of the association between mild flu and IFITM3 rs12252 polymorphisms, BMI, diabetes, and hypercholesterolemia in an Iranian sample with Fars ethnic background: observed powers. (DOCX 20 kb) [file 12985_2017_884_MOESM2_ESM.docx]

**Additional file 2.** Univariable and multivariable logistic regression of the association between mild flu and *IFITM3* rs12252 polymorphisms, BMI, diabetes, and hypercholesterolemia in an Iranian sample with Fars ethnic background: observed powers

**Table A: Power analysis results of the univariable logistic regression**

| **Variables** | **Case vs. control** |  |  |  |
| --- | --- | --- | --- | --- |
|  | **OR** | **95% CI** | ***P* value** | **Observed Power** |
| **Age** _(mean ± SD)_ | 0.99 | 0.98, 1.00 | 0.275 | 0.84 |
| **BMI** _(mean ± SD)_ | 1.06 | 1.01, 1.13 | 0.034 | 0.79 |
| **Province** |  |  |  |  |
| Zanjan | 1 | - | - |  |
| Semnan | 0.36 | 0.13, 0.95 | 0.039 | 0.70 |
| Markazi | 1.19 | 0.42, 3.36 | 0.745 | 0.68 |
| **Hypercholesterolemia** _n (%)_ |  |  |  |  |
| Positive | 0.27 | 0.08, 0.96 | 0.042 | 0.61 |
| Negative | 1 | - | - |  |
| **Diabetes** _n (%)_ |  |  |  |  |
| Positive | 0.35 | 0.17, 0.73 | 0.005 | 0.94 |
| Negative | 1 | - | - |  |
| **Genotype** _n (%)_ |  |  |  |  |
| CC | 5.37 | 0.55, 52.68 | 0.149 | 0.69 |
| TC | 4.03 | 1.20, 13.58 | 0.025 | 0.89 |
| CC+CT | 4.29 | 1.45, 12.72 | 0.008 | 0.89 |
| TT | 1 | - | - |  |
| **Allele** _n (%)_ |  |  |  |  |
| C allele | 4.26 | 4.27 | 0.0016 | 0.81 |
| T allele | 1 | - | - |  |

**Table B:** Power analysis results of the multivariable logistic analysis

| **Genotype** | **Final model** | **95% CI** | ***P* value** | **Observed Power** |
| --- | --- | --- | --- | --- |
| CC | 2.71 | 0.26, 28.57 | 0.406 | 0.65 |
| CT | 7.62 | 1.69, 34.39 | 0.008 | 0.67 |
| CC+CT | 5.92 | 1.59, 22.09 | 0.007 | 0.68 |
| TT | 1 | - | - |  |
